# Impact of the COVID-19 pandemic on acute coronary syndrome hospital admission and management in Slovenia

**DOI:** 10.1136/openhrt-2023-002440

**Published:** 2023-11-20

**Authors:** Tjaša Furlan, Janez Bijec, Petra Došenović Bonča, Irena Ograjenšek, Borut Jug

**Affiliations:** 1 Department of Internal Medicine, General Hospital Trbovlje, Trbovlje, Slovenia; 2 Faculty of Electrical Engineering, University of Ljubljana, Ljubljana, Slovenia; 3 School of Economics and Business, University of Ljubljana, Ljubljana, Slovenia; 4 Department of Vascular Disease, University Clinical Centre Ljubljana, Ljubljana, Slovenia

**Keywords:** COVID-19, acute coronary syndrome, global health

## Abstract

**Aims:**

We evaluated the effects of the COVID-19 pandemic on hospital admission and quality of care for acute coronary syndrome.

**Methods and results:**

Data for all patients admitted to hospital care for acute coronary syndromes in Slovenia (nationwide cohort) between 2014 and 2021 were obtained by merging the national hospital database, national medicines reimbursement database and population mortality registry using unique identifying numbers. Using interrupted time series analysis, we assessed the impact of the COVID-19 pandemic on hospital admission rates and quality of care (in-hospital and 30-day mortality, reperfusion and secondary preventive medication uptake). Data were fitted to segmented regression models with March 2020 as the breakpoint. Data on 21 001 patients were included (7057 ST-elevation myocardial infarction (STEMI), 7649 non-ST elevation myocardial infarction (NSTEMI) and 6295 unstable angina). Hospital admissions for STEMI remained stable (92 patients; +1 patient per month, p=0.783), whereas the pandemic was associated with a significant reduction in NSTEMI (81 patients; −21 patients per month, p=0.015) and unstable angina admissions (47 patients; −28 patients per month, p=0.025). In patients with STEMI, the pandemic did not affect reperfusion rates (0.29%, (95% CI) −1.5% to 2.1%, p=0.755) or in-hospital mortality (0.1%, (95% CI) −0.9% to 1.1%, p=0.815), but was associated with a significant negative trend for secondary preventive medication uptake (−0.12%, (95% CI) −0.23% to −0.01%, p=0.034).

**Conclusion:**

In Slovenia, hospital admissions for STEMI remained stable throughout the COVID-19 pandemic, but NSTEMI and unstable angina admissions dropped significantly. While mortality and reperfusion rates were not affected, the pandemic was associated with a continual negative time trend for the uptake of secondary preventive medication.

**WHAT IS ALREADY KNOWN ON THIS TOPIC**

- The COVID-19 pandemic has severely disrupted the provision of acute cardiac care, including the effective management of acute coronary syndromes.
- Several reports suggest a drop in hospital admissions for myocardial infarction and revascularisation procedures after the outbreak of the COVID-19 pandemic

**WHAT THIS STUDY ADDS**

- The COVID-19 pandemic has significantly affected hospital admission patterns for acute coronary syndromes: while ST-elevation myocardial infarction (STEMI) admissions remained stable, non-ST elevation myocardial infarction (NSTEMI) and unstable angina admission rates dropped markedly following the pandemic outbreak.
- Moreover, while acute cardiac care measures for STEMI (ie, mortality and reperfusion rates) have not been affected by the pandemic, a negative trend in secondary preventive medication uptake suggests reduced quality of long-term care for STEMI.

**HOW THIS STUDY MIGHT AFFECT RESEARCH, PRACTICE OR POLICY**

- Our findings show that the pandemic resulted in under-diagnosis and/or under-management of patients with acute coronary syndromes, which should be intensively addressed in the postpandemic period.

## Introduction

Coronary artery disease remains a significant cause of morbidity and mortality worldwide despite continual advances in managing acute and chronic coronary syndromes.^1^ Current guidelines suggest acute reperfusion (addressing the immediate risk of myocardial complications and mortality) and long-term secondary prevention (addressing the lifelong risk of atherosclerosis progression and events) as two pivotal evidence-based disease-modifying approaches for the management of patients presenting with acute coronary syndromes.^2–4^ Therefore, the uptake of reperfusion therapy and secondary prevention medication have been proposed as valid, reliable and meaningful indicators of care for patients with acute coronary syndromes.^5^

However, the COVID-19 pandemic has severely disrupted the provision of acute cardiac care, including the effective management of acute coronary syndromes. Several reports suggest a drop in hospital admissions for myocardial infarction and revascularisation procedures after the outbreak of the COVID-19 pandemic.^6–8^ The regions most affected by the pandemic reported a significant reduction in admissions for acute myocardial infarction, with a parallel increase in mortality and complication rates.^9–11^ Still, the extent of the pandemic-associated disruption in service provision and quality of care across different subsets of coronary artery disease patients, countries or phases of the pandemic remains to be appraised.

In the present study, we sought to evaluate the effects of the COVID-19 pandemic on hospital admission and management of acute coronary syndromes in Slovenia. We hypothesised that the COVID-19 pandemic would reduce hospital admissions and quality of care, followed by a gradual recovery towards prepandemic levels. Using interrupted time series analysis, we appraised the change in the level and trend of hospital admissions and quality of care (in-hospital and 30-day mortality, reperfusion procedures and secondary preventive medication uptake) following the COVID-19 pandemic outbreak in Slovenia.

## Methods

### Data source and patient involvement

We conducted a nationwide analysis of the effect of the COVID-19 pandemic on hospital admission and quality of care of acute coronary syndrome using an interrupted time series framework.

We included all patients admitted to hospital inpatient care in Slovenia for acute coronary syndromes (ICD,International Classification of Diseases codes I20 and I21) between 10 October 2014 and 30 June 2021. Acute coronary syndromes were further categorised into (1) ST-elevation myocardial infarction (STEMI; ICD codes I21.0-I21.3), (2) non-ST elevation myocardial infarction (NSTEMI; ICD code I21.4) or (3) unstable angina pectoris (ICD code I20.0).

Using unique personal identifiers, we linked patients’ information from (1) the Hospital Discharge Statistics Database, (2) the National Mortality Registry and (3) the Medication Reimbursement Database, which are routinely collected at The Health Insurance Institute of Slovenia and represent a comprehensive nationwide population. We excluded patients with subsequent hospital admission for acute coronary syndrome and patients without health insurance ID numbers (eg, tourists and visitors).

The reported data include (1) patients’ baseline characteristics (age, sex, discharge diagnoses of arterial hypertension, dyslipidaemia, diabetes mellitus, peripheral artery disease, cerebrovascular disease, heart failure, atrial fibrillation, dementia, depression, chronic obstructive pulmonary disease or asthma and the total number of coded concomitant comorbidities) and (2) acute setting management with posthospitalisation care (reperfusion procedure during hospitalisation, secondary preventive medication uptake and in-hospital and 30-day mortality).

### Hospital management and outcomes

We analysed pre-COVID-19 and post-COVID-19 time trends for hospital admission and quality of hospital care indicators: (1) reperfusion procedure (percentage of patients with a reperfusion procedure during hospital stay), (2) secondary preventive medication uptake (percentage of patients that made a reimbursement claim for dual antiplatelet therapy/anticoagulation, beta-blocker, lipid-lowering therapy and ACE inhibitor/angiotensin receptor blocker within 30 days after discharge, restricted to patients who were alive 30 days after admission, and based on anatomic therapeutic classification codes) and (3) mortality (in-hospital mortality and 30-day mortality after discharge).

Outcomes data were aggregated over monthly time units (ie, the proportion of patients meeting selected quality of hospital care indicators per month).

### Statistical analysis

Statistical analyses were performed with Stata V.17.0 for Mac (2017, StataCorp). Data were appraised for normality of distribution using the Kolmogorov-Smirnov test and the Shapiro-Wilk test for the aggregated time series data.

The baseline characteristics of the study population were compared using descriptive statistics. Continuous data were summarised as means (with SD) for normally distributed or medians (with IQR) for non-normally distributed continuous variables. Categorical data were summarised as counts and percentages. Normally distributed continuous variables were compared using t-test, non-normally distributed continuous variables using the Mann-Whitney U test and categorical variables using χ^2^ test. Logistic regression models were used to adjust the probability of the outcome for the differences in baseline characteristics.^12^ The model covariates included age, sex, the total number of comorbidities, total number of procedures during hospitalisation, discharge diagnoses coded as atrial fibrillation (I48), heart failure (I50-I50.9), arterial hypertension (I10, I11-I11.9, I12-I12.9, I13-I13.9), diabetes mellitus (E09.21-E09.9, E10-E10-E10.9, E11-E11.9, E13.01-E13.9, E14-E14.9, E15, E16-E16.9), chronic kidney disease (N10, N17-N17.9, N18-N18.9, N19), dementia (F00.0-F00.9, F01-F01.9, F02.3, F02.8, F03, F05.1), depression (F06.4, F32-F32.91, F33.0-F33.9) and chronic obstructive pulmonary disease or asthma (J44.0-J44.9, J45-J45.9, J46). Statistical significance was set at two-tailed p<0.05.

### Interrupted time series analysis

The data were fitted to segmented regression models for interrupted time series.^13^

Model assumptions were assessed with correlograms (autocorrelation and partial autocorrelation function) and residual plots and adjusted as appropriate (eg, differencing for stationarity, dummy variables for seasonality and/or autoregressive orders).

Segmentation was based on the stringency index, which is a composite measure based on nine response indicators, including school closures, workplace closures, cancellation of public events, restrictions on public gatherings, closures of public transport, stay-at-home requirements, public information campaigns, restrictions on internal movements and international travel controls.^14^ The index on any given day is calculated as the mean score of the nine metrics, each taking a value between 0 and 100 (100=strictest). Based on the stringency index, 30 March 2020 was determined as the time series breakpoint. Because our outcomes data were aggregated over monthly time units, we selected March 2020 as the breakpoint.

## Results

### Baseline characteristics

Between 10 October 2014 and 30 June 2021, 21 001 patients met the inclusion criteria. We included 7057 patients with STEMI, 7649 with NSTEMI and 6295 patients with unstable angina. The number of STEMI hospital admissions has been declining pre pandemic and continued to decrease on the level and trend after the breakpoint. Conversely, NSTEMI and especially unstable angina hospital admission dropped significantly; while NSTEMI admissions recovered to prepandemic rates within the following 2 years, unstable angina admissions remained below prepandemic levels (online supplemental material).

10.1136/openhrt-2023-002440.supp1 Supplementary data

10.1136/openhrt-2023-002440.supp2 Supplementary data

Despite uninterrupted time trends in STEMI admissions, patients hospitalised after 1 March 2020, breakpoint had significantly fewer co-morbidities while remaining comparable in age and sex (table 1).

**Table 1:** Baseline demographics for pre-COVID-19 and post-COVID-19 group of patients with STEMI

	Pre-COVID-19 group (before March 2020) n=5689	Post-COVID-19 group (after March 2020) n=1368	P value
Age, mean (SD)	65.5 (13.3)	64.9 (13.1)	0.148
Male, %	67.5	68.3	0.607
Atrial fibrillation, %	11.2	8.8	0.011
Heart failure, %	13.9	11.4	0.017
Arterial hypertension, %	54.6	46.0	0.000
Diabetes mellitus, %	19.3	17.0	0.054
Chronic kidney disease, %	8.5	7.9	0.514
COPD/asthma, %	4.1	3.4	0.250
Dementia, %	1.9	0.9	0.007
Depression, %	0.8	0.5	0.471
Cancer, %	2.8	1.6	0.008

COPD, chronic obstructive pulmonary disease; n, number of patients; STEMI, ST-elevation myocardial infarction.

### Hospital management and outcomes

#### Hospital admission

**Figure 1:**
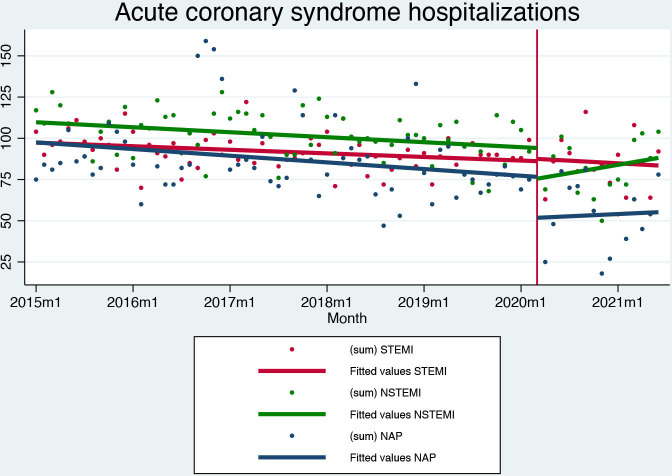
Interrupted time series analysis for acute coronary syndrome hospitalisations. NAP, non-stable angina pectoris; NSTEMI, non-ST elevation myocardial infarction; STEMI, ST-elevation myocardial infarction.

The COVID-19 outbreak caused no significant change in the monthly number of patients hospitalised for STEMI (91 patients; +1 patient per month, p=0.783). We observed a substantial decrease in the monthly number of patients hospitalised for NSTEMI (81 patients; −21 patients per month, p=0.015) and unstable angina (47 patients; −28 patients per month, p=0.025) (figure 1).

Given the stability of STEMI admissions, we further analysed the quality of care in patients with STEMI.

#### Reperfusion procedures

**Figure 2:**
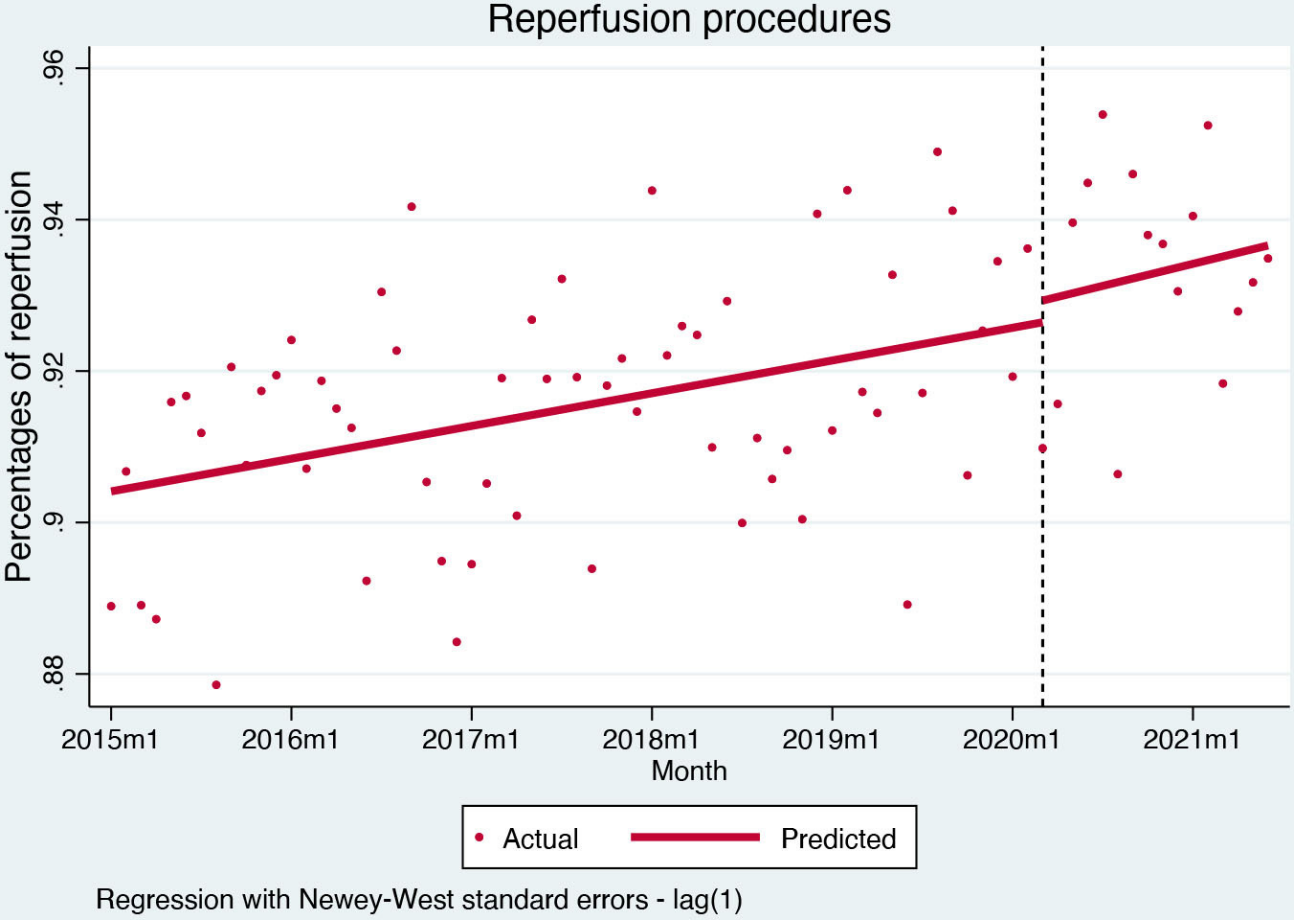
Interrupted time series analysis for reperfusion procedures.

The proportion of STEMI patients treated with a reperfusion procedure during hospitalisation at the start of observation was 90% and was increasing (0.04% per month, (95% CI) 0.02% to 0.06%, p=0.001). After the pandemic outbreak, the proportion of patients with reperfusion procedures did not change significantly (0.29%, (95% CI) −1.5% to 2.1%, p=0.755) (figure 2).

#### Discharge medication of secondary prevention

**Figure 3:**
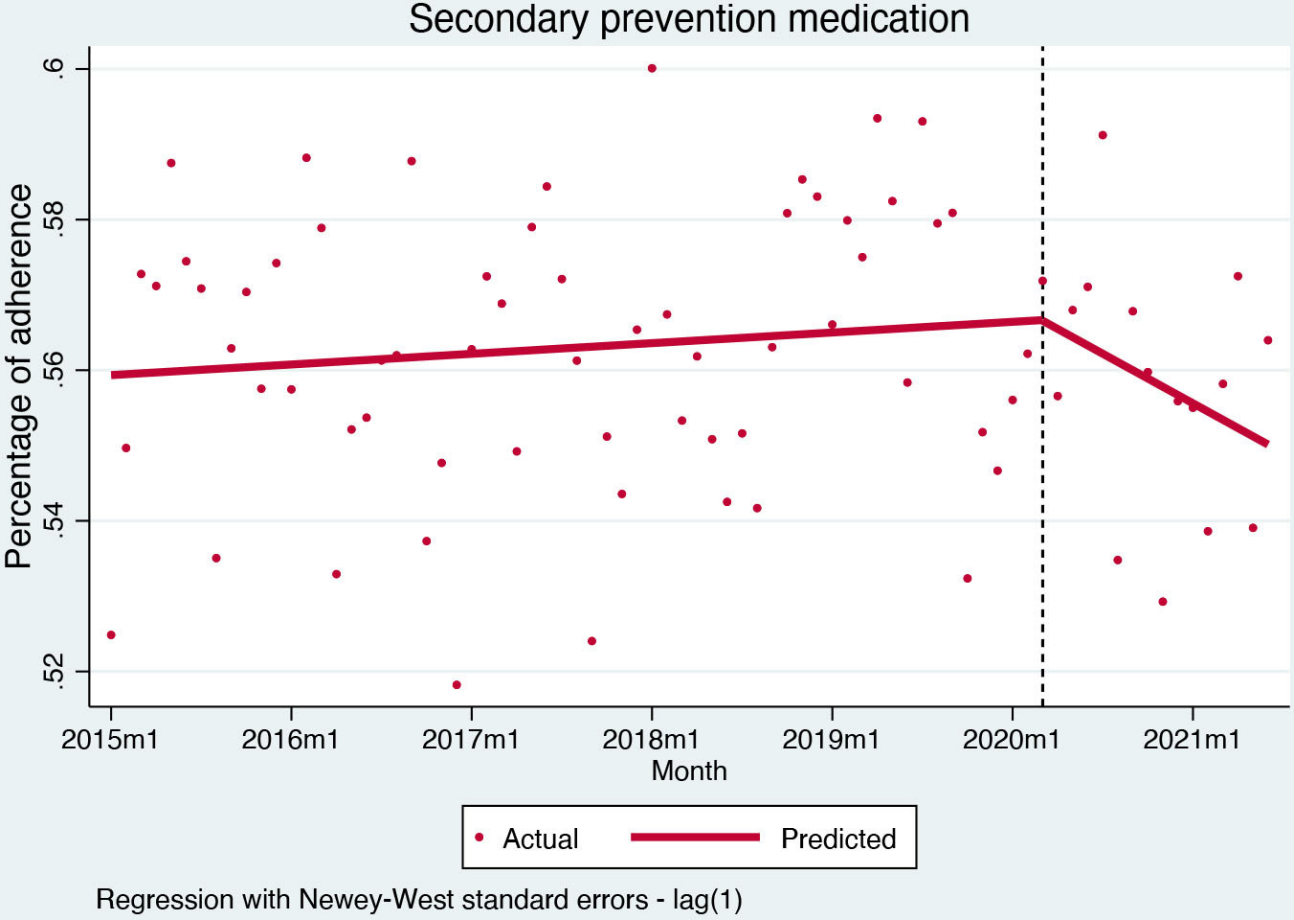
Interrupted time series analysis for secondary prevention medication 30 days after discharge.

Uptake of secondary preventive medication was stable before the pandemic (0.012% per month, (95% CI) −0.02% to 0.04%, p=0.440). After the pandemic outbreak, there was no significant change in the level of secondary preventive medication uptake (−0.008%, (95% CI) −1.53% to 1.51%, p=0.991), whereas the trend became negative (−0.12%, (95% CI) −0.23% to −0.01%, p=0.034) (figure 3). There was also no change in antiplatelet therapy or antihyperlipidemic therapy alone at the start of the pandemic (antiplatelet therapy: 0.03%, (95% CI) −0.99% to 1.05%, p=0.953; antihyperlipidemic treatment: 0.63%, (95% CI) −0.81% to 2.08%, p=0.384), but there was a negative trend after that (antiplatelet therapy: −0.08%, (95% CI) −0.15% to −0.01%, p=0.028; antihyperlipidemic therapy: −0.09%, (95% CI) −0.18% to 0.01%, p=0.066).

#### Mortality

**Figure 4:**
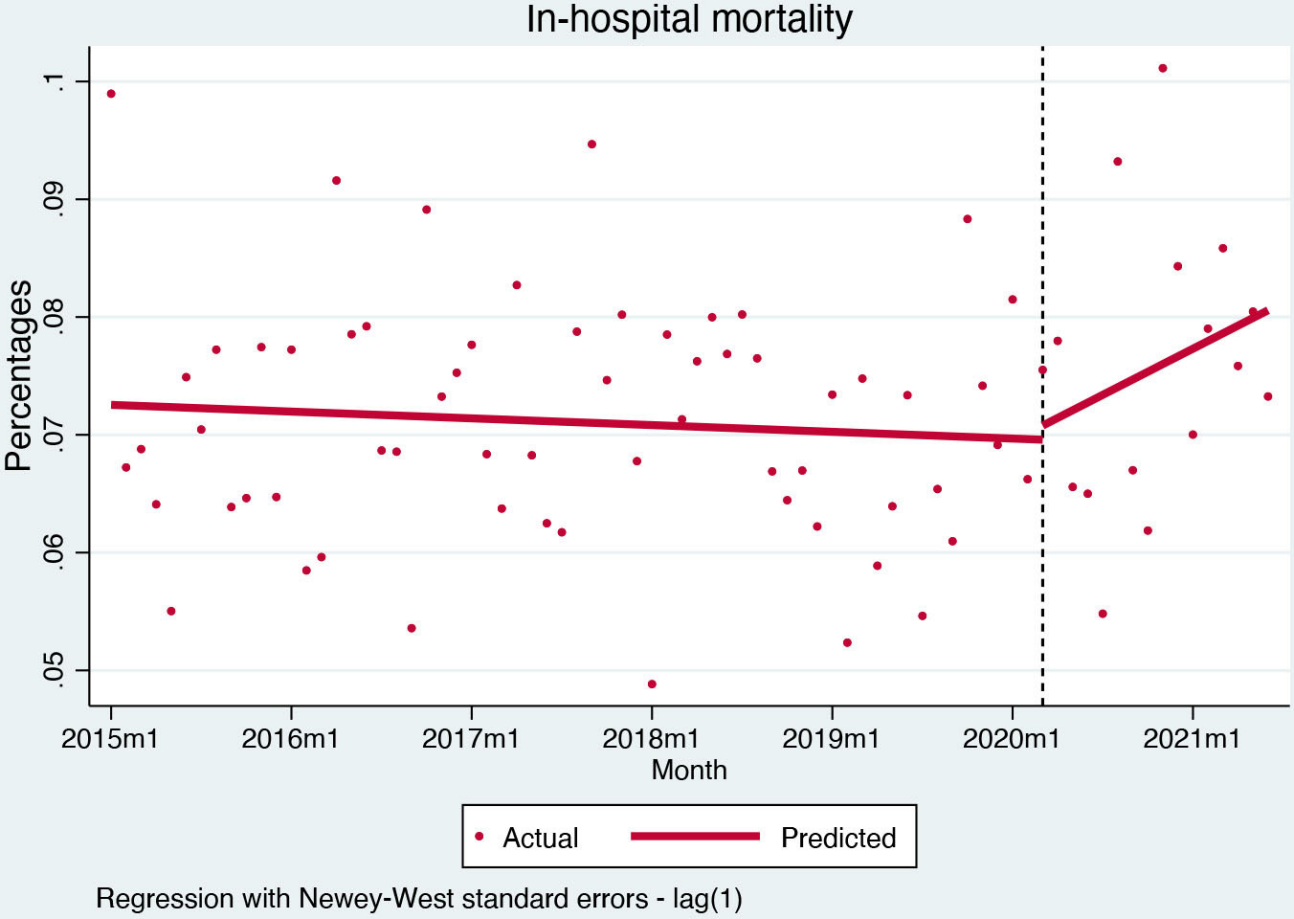
Interrupted time series analysis for in-hospital mortality.

**Figure 5:**
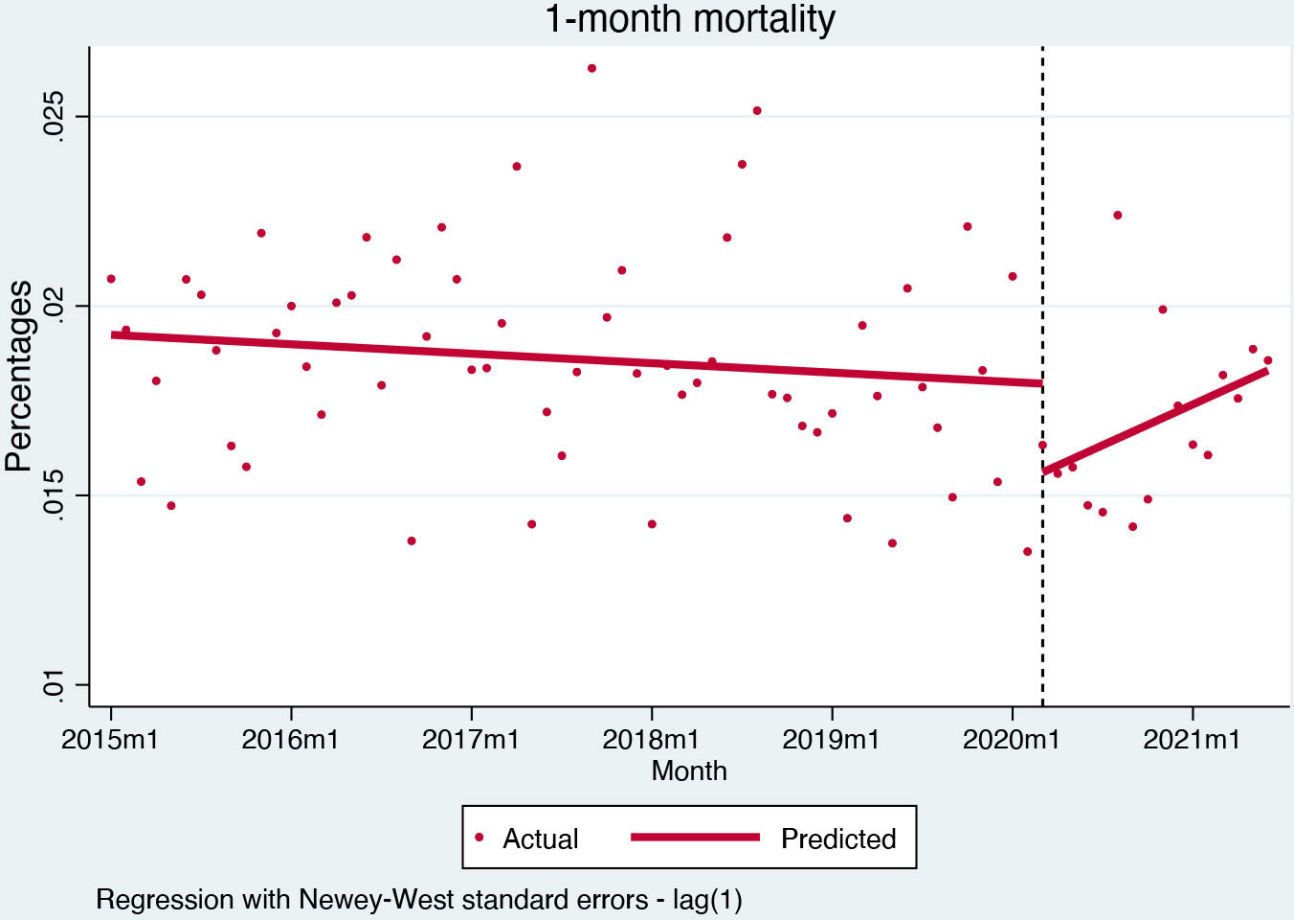
Interrupted time series for 1-month mortality.

In-hospital mortality was stable before the pandemic (−0.005% per month, (95% CI) −0.02% to 0.01%, p=0.521). After the pandemic outbreak, there was no change in in-hospital mortality (0.1%, (95% CI) −0.9% to 1.1%, p=0.815) (figure 4). One-month mortality was also stable before the pandemic (−0.002% per month, (95% CI) −0.006% to 0.001%, p=0.246), but the pandemic outbreak caused a slight but significant decrease (−0.23%, (95% CI) −0.44% to −0.03%, p=0.025) (figure 5).

## Discussion

The COVID-19 pandemic severely affected the presentation of acute coronary syndromes in Slovenia. STEMI admissions remained stable, with reperfusion and mortality rates unaffected by the course of the pandemic, but a significant negative trend in secondary preventive medication uptake—suggesting an effect on the quality of care post-hospitalisation. Conversely, hospital admissions for NSTEMI and unstable angina dropped significantly. While NSTEMI admissions display a sharp drop in level but a gradual recovery with a positive trend for hospital admissions after the pandemic outbreak, unstable angina admissions dropped and remained low throughout the pandemic period.

Our results align with previous reports on pandemic-associated disruption of acute cardiac care, primarily attributed to the diversion of healthcare resources and the measures to contain the pandemic, including fear of contagion and social distancing guidance.^15^ Other studies have shown a decline in hospitalisation for all types of acute myocardial infarction,^9–11^ especially in the regions most affected by the pandemics.^9^ Conversely, our analysis suggests a differential effect of the pandemic on different subtypes of acute coronary syndromes, which likely reflects the specific clinical pathways for diagnosis and management of STEMI versus non-STEMI acute coronary syndromes. While STEMI can be readily diagnosed with clinical presentation and ECG within every healthcare community centre in Slovenia (and a fast-track transfer to a catheterisation laboratory), NSTEMI and unstable angina require a more comprehensive approach, including troponin assessment and hours-long waiting time within already overwhelmed secondary emergency department facilities,^16^ which may likely have diverted referrals. Even studies showing a significant reduction in all coronary artery disease hospitalisations suggest a more profound decrease in non-STEMI admissions.^8 9^ Possible reasons notwithstanding, the marked drop in non-STEMI acute coronary syndromes during the pandemic is troublesome; given the high mortality associated with the natural course of non-STEMI acute syndromes,^17^ we hypothesise that such a drop in acute hospital care for NSTEMI and unstable angina patients may partially explain the excess mortality observed throughout the pandemic.^18^

Regarding STEMI acute care, adjusted mortality and reperfusion rates remained unaffected by the pandemic in Slovenia. Importantly, mortality rates—in-hospital and 30-day—for STEMI remained stable throughout the pandemic. While previous studies showing increased mortality rates^11^ also reported lower reperfusion rates,^19 20^ we observed no change in mortality or reperfusion procedures. The finding can be explained by specific responses of different healthcare systems to the pandemic; in Slovenia, despite the severe redistribution of resources and skilled personnel, primary percutaneous intervention for myocardial infarction remained operational throughout the pandemic and the small geographic area permitted rapid transfers to and from facilities providing 24/7 acute cardiac care. It is also noteworthy that STEMI patients during the pandemic had fewer recorded comorbidities when compared with the prepandemic STEMI population; this may be attributable to a lower propensity of physicians to record all comorbidities during the pandemic-related diversion of resources and personnel but might also suggest that some patients with comorbidities were reluctant to seek medical attention because of fear of contagion.

Regarding STEMI secondary preventive care, medication uptake—while not immediately affected by the outbreak—displays an unfavourable trend throughout the pandemic. While reperfusion therapies strongly reflect the quality of STEMI hospital care, secondary preventive medication uptake indicates a successful transition to complex long-term management and is more likely affected by the pandemic. This is corroborated by a gradual downtrend throughout the pandemic rather than a significant drop when the pandemic broke out. Our results align with previous reports: uptake, for instance, was not affected by the pandemic.^21^ Similarly, in our study, we did not observe an expected increase in antihyperlipidemic therapy—despite statins being promoted during the pandemic for their possible associations with improved COVID-19 outcomes.^22^

An interesting finding in our results is that the patients hospitalised for STEMI during the pandemic had fewer comorbidities. Hence, even in the STEMI group, only healthier and low-risk patients were likely to be hospitalised. On the one hand, even though we used adjusted mortality rates, unmeasured confounders may have directed the lower-than-expected mortality. On the other hand, patients with more comorbidities might be missed due to out-of-hospital cardiac arrest.^23^

### Limitations

Our study has some limitations. First, this was a national administrative health database-driven observational study with all inherent biases that may derive from such study design.^13^ While mortality, reperfusion rates and secondary preventive medication uptake were adjusted for patient-level factors, such as recorded comorbidities, unmeasured confounders (eg, obesity, metabolic syndrome, smoking) may have biased our results. Second, time series analysis was performed on aggregated data and cannot provide individual patient inference. Third, our data pertains to patients admitted for acute coronary syndromes in Slovenia; our findings cannot infer out-of-hospital events or be straightforwardly generalised to other healthcare systems. Nevertheless, ours was a population-wide study (ie, encompassing all patients hospitalised for acute coronary syndromes in the country), thus providing reliable data on hospital admission trends, quality of care and outcomes.

### Conclusions

Our results show that the COVID-19 epidemic has significantly affected hospital admission patterns for acute coronary syndromes: while STEMI admissions remained stable, NSTEMI and unstable angina admission rates dropped markedly following the pandemic outbreak. Moreover, while acute cardiac care measures for STEMI (ie, mortality and reperfusion rates) have not been affected by the pandemic, a negative trend in secondary preventive medication uptake suggests reduced quality of long-term care for STEMI. Our findings show that the pandemic resulted in under-diagnosis and/or under-management of patients with acute coronary syndromes, which should be intensively addressed in the postpandemic period.

## Data Availability

Data are available upon reasonable request.
