# Supplementary material for: Impact of the COVID-19 pandemic on acute coronary syndrome hospital admission and management in Slovenia
Source: Open Heart. 2023 Nov 20;10(2):e002440. doi: 10.1136/openhrt-2023-002440 (PMC10660426; doi:10.1136/openhrt-2023-002440)
Supplement: Supplementary data [file openhrt-2023-002440supp001.pdf]

APPENDICES

Appendix 1. Impact of COVID-19 on hospitalization and quality of care for STEMI, NSTEMI, and unstable angina.

	Monthly change before the COVID-19 pandemic		COVID-19 pandemic (March 2020)		Monthly change after the COVID-19 pandemic	
	incidence/100.000 people or %	p	incidence/100.000 people or %	p	incidence/100.000 people or %	p
STEMI						
hospitalizations	-0.009, [95%CI] -0.01, -0.003	0.002	0.08, [95%CI] -0.51, 0.68	0.783	-0.004, [95%CI] -0.07, 0.06	0.907
reperfusion procedures	0.04%, [95%CI] 0.02, 0.06	0.001	0.29%, [95%CI] -1.5, 2.1	0.755	0.01%, [95%CI] -0.2, 0.2	0.878
secondary prevention medication	0.01%, [95%CI] -0.02, 0.04	0.440	-0.01%, [95%CI] -1.5, 1.5	0.991	-0.12%, [95%CI] -0.2, -0.01	0.034
in-hospital mortality	-0.005%, [95%CI] -0.02, 0.01	0.521	0.12%, [95%CI] -0.9, 1.1	0.815	0.07%, [95%CI] -0.01, 0.15	0.079
1-month mortality	-0.002%, [95%CI] -0.01, 0.001	0.246	-0.23%, [95%CI] -0.44, -0.03	0.025	0.02%, [95%CI] 1.8, 2.0	0.001
NSTEMI						
hospitalizations	-0.011, [95%CI] -0.02, -0.002	0.017	-0.91, [95%CI] -1.64, -0.18	0.015	0.05, [95%CI] -0.02, 0.12	0.163
reperfusion procedures	0.02%, [95%CI] -0.02, 0.06	0.370	-0.19%, [95%CI] -3.7, 3.2	0.914	-0.07%, [95%CI] -0.45, 0.32	0.731
secondary prevention medication	0.01%, [95%CI] -0.01, 0.03	0.337	0.16%, [95%CI] -1.4, 1.8	0.846	-0.08%, [95%CI] -0.25, 0.1	0.378
in-hospital mortality	-0.001%, [95%CI] -0.01, 0.01	0.787	0.3%, [95%CI] -0.14, 0.73	0.175	0.001%, [95%CI] -0.05, 0.05	0.962
1-month mortality	-0.003%, [95%CI] -0.007, 0.001	0.122	0.21%, [95%CI] -0.04, 0.47	0.103	-0.002%, [95%CI] 2.1, 2.4	0.935
Unstable angina						
hospitalizations	-0.01, [95%CI] -0.03, -0.001	0.041	-1.26, [95%CI] -2.35, -0.16	0.025	0.02, [95%CI] -0.07, 0.12	0.610
reperfusion procedures	-0.02%, [95%CI] -0.02, 0.01	0.001	0.85%, [95%CI] 0.01, 1.7	0.046	-0.04%, [95%CI] -0.1, 0.04	0.318
secondary prevention medication	-0.08%, [95%CI] -0.1, -0.07	0.000	2.3%, [95%CI] -0.1, 4.8	0.060	-0.02%, [95%CI] -0.3, 0.25	0.863
in-hospital mortality	-0.002%, [95%CI] -0.01, 0.01	0.589	-0.17%, [95%CI] -0.57, 0.22	0.388	-0.002%, [95%CI] -0.03, 0.02	0.858
1-month mortality	-0.001%, [95%CI] -0.003, 0.0002	0.093	-0.05%, [95%CI] -0.13, 0.02	0.145	0.001%, [95%CI] -0.01, 0.01	0.812

STEMI – ST-elevation myocardial infarction, NSTEMI – non-ST-elevation myocardial infarction
