# Supplementary figures and images for: Impact of the COVID-19 pandemic on acute coronary syndrome hospital admission and management in Slovenia
Source: Open Heart. 2023 Nov 20;10(2):e002440. doi: 10.1136/openhrt-2023-002440 (PMC10660426; doi:10.1136/openhrt-2023-002440)

# ACUTE CORONARY SYNDROME during the COVID-19 pandemic

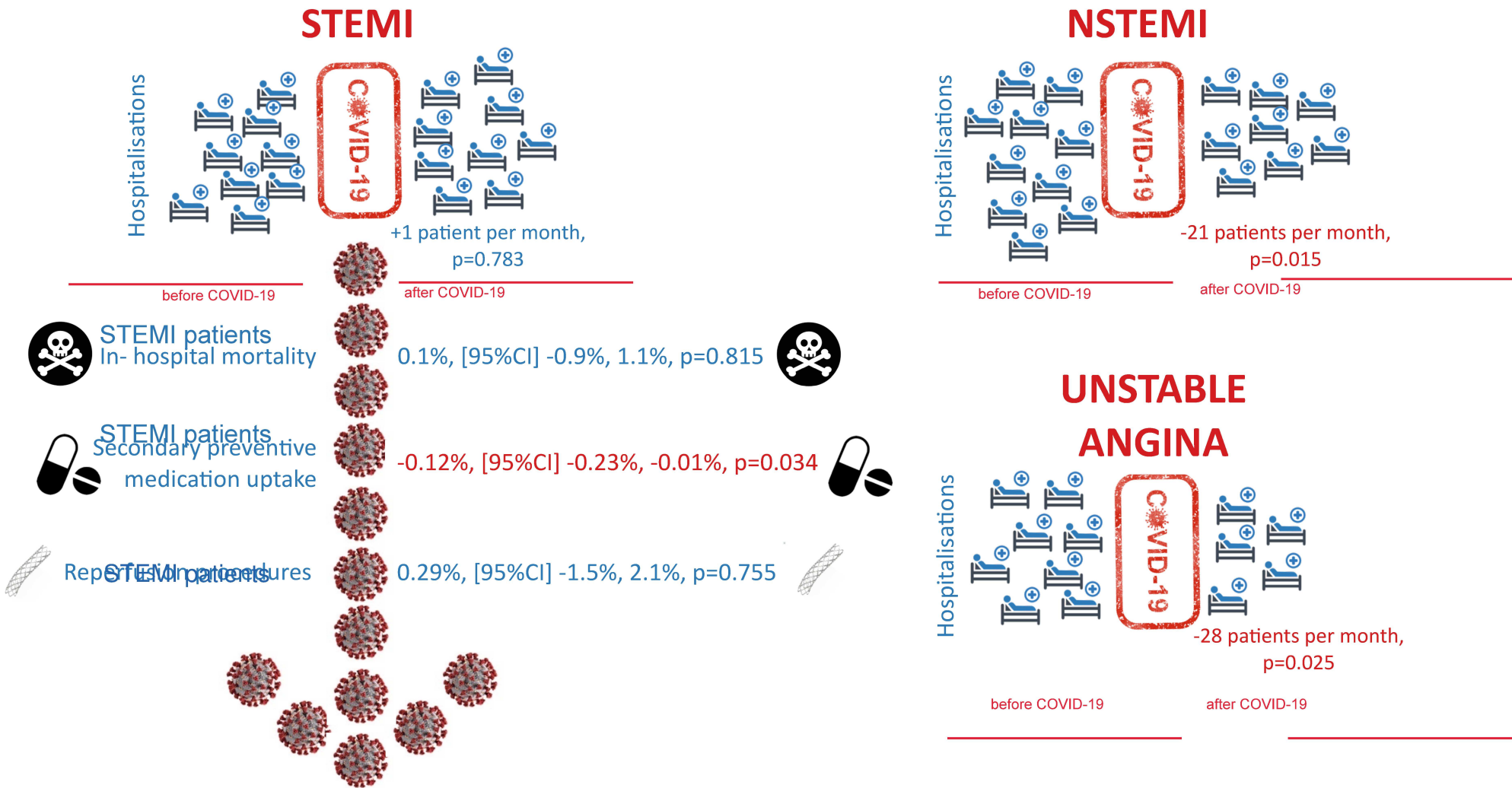

Supplement: Supplementary data [file openhrt-2023-002440supp002.pdf]
